# Evidence for *Bartonella quintana* in Lice Collected from the Clothes of Ethiopian Homeless Individuals

**DOI:** 10.3390/pathogens12111299

**Published:** 2023-10-30

**Authors:** Tafese Beyene Tufa, Gabriele Margos, Volker Fingerle, Christine Hartberger, Sven Poppert, Richard J. Birtles, Peter Kraiczy, Volkhard A. J. Kempf, Hagen Frickmann, Torsten Feldt

**Affiliations:** 1 Asella Teaching and Referral Hospital, College of Health Sciences, Arsi University, Asella P.O. Box 04, Ethiopia; tafeseb.tufa@yahoo.com; 2 Hirsch Institute of Tropical Medicine (HITM), Heinrich-Heine University, Asella P.O. Box 04, Ethiopia; 3 Department of Gastroenterology, Hepatology and Infectious Diseases, University Medical Center Düsseldorf, 40225 Düsseldorf, Germany; 4 National Reference Center for Borrelia, Bavarian Health and Food Safety Authority (LGL), Branch Oberschleißheim, 85764 Oberschleißheim, Germany; gabriele.margos@lgl.bayern.de (G.M.); volker.fingerle@lgl.bayern.de (V.F.); christine.hartberger@lgl.bayern.de (C.H.); 5 Diagnostic Department, Bernhard Nocht Institute for Tropical Medicine Hamburg, 20239 Hamburg, Germany; sven@poppert.eu; 6 School of Science, Engineering and Environment, University of Salford, Salford M5 4WT, UK; r.j.birtles@salford.ac.uk; 7 Institute for Medical Microbiology and Infection Control and Consiliary Laboratory for Bartonella Infections (Appointed by the Robert Koch Institute), University Hospital, Goethe University Frankfurt, 60596 Frankfurt am Main, Germany; kraiczy@em.uni-frankfurt.de (P.K.); volkhard.kempf@kgu.de (V.A.J.K.); 8 Department of Microbiology and Hospital Hygiene, Bundeswehr Hospital Hamburg, 20359 Hamburg, Germany; 9 Institute for Medical Microbiology, Virology and Hygiene, University Medicine Rostock, 18057 Rostock, Germany

**Keywords:** Ethiopia, xenosurveillance, *Pediculus humanus*, *Bartonella quintana*, infection risk, vector

## Abstract

Human lice, *Pediculus humanus*, can transmit various pathogens, including *Bartonella quintana*, *Borrelia recurrentis*, and *Rickettsia prowazekii*. Xenosurveillance is an epidemiological approach to assessing human infection risks performed by screening vectors of infectious disease agents. In the proof-of-principle study reported herein, the DNA of 23 human lice was collected from the clothes of 30 homeless Ethiopian individuals. These samples were assessed using 16S rRNA gene-specific pan-eubacterial PCR for screening, followed by *Bartonella* genus 16S-23S internal transcribed spacer (ITS) sequence-specific PCR, *Bartonella* genus *gltA* gene-specific PCR, and 16S rRNA gene PCR with specificity for relapsing-fever-associated *Borrelia* spp. with subsequent sequencing of the amplicons. In one sample, the pan-eubacterial 16S rRNA gene-specific screening PCR, the *Bartonella* genus 16S-23S ITS sequence-specific PCR, and the *Bartonella* genus *gltA* gene-specific PCR allowed for the sequencing of *B. quintana*-specific amplicons. In two additional samples, *Bartonella* genus *gltA* gene-specific PCR also provided sequences showing 100% sequence identity with *B. quintana*. In total, 3/23 (13.0%) of the assessed lice were found to be positive for *B. quintana*. Correlating clinical data were not available; however, the assessment confirmed the presence of *B. quintana* in the local louse population and thus an associated infection pressure. Larger-sized cross-sectional studies seem advisable to more reliably quantify the infection risk of lice-infested local individuals. The need for prevention by providing opportunities to maintain standard hygiene for Ethiopian homeless individuals is stressed by the reported findings, especially in light of the ongoing migration of refugees.

## 1. Introduction

If standard hygiene precautions cannot be maintained in cases of war, crisis, or displacement, there is an increased risk of the spread of human ectoparasites. Next to causing mechanically induced discomfort, several human ectoparasites have also been associated with the transmission of agents causing infectious diseases. For human lice, their role as vectors for harmful pathogens like *Borrelia recurrentis*, *Rickettsia prowazekii*, and *Bartonella quintana* is considered well established [1,2,3,4,5,6,7,8]. Regarding *Yersinia pestis*, the causative agent of bubonic plague, the possibility of human lice playing a vector role is still controversial but considered likely based on epidemiological and laboratory-based evidence [1]. Even bacteria primarily linked to nosocomial spread like *Acinetobacter baumannii* have recently been associated with louse transmission among humans [9,10].

The human louse, *Pediculus humanus*, comprises the ecotypes head louse and body louse. The molecular discrimination of the subtypes of this louse based on mitochondrial clades A-F is feasible [11]. While it had formerly been assumed that pathogen transmission was basically restricted to body lice, this potential has recently been shown to apply to head lice as well. The effectiveness of preventive medical approaches is hampered by complex epigenetic control of gene expression, which causes a considerable level of flexibility of reaction towards altered environmental conditions, including the acquisition of ivermectin resistance [11,12]. In biting events, a potent vasodilator is known to be injected into the human host [13]. Cold weather and lack of hygiene, e.g., factors applicable to homeless individuals, facilitate the spread of human lice [14,15].

The occasional importation of *B. recurrentis*, the causative agent of louse-borne relapsing fever [16,17,18], to Europe in the course of recent migration movements has increased the awareness of this otherwise poorly known pathogen in European health centers [19,20]. The frequently reported symptoms comprise fever, headache, jaundice, epistaxis, and hepatosplenomegaly [21]. Recent reports have shown that epidemiological data published on louse-borne relapsing fever are highly variable; in particular, severe outcomes like death either due to the disease itself or a therapy-associated Jarisch–Herxheimer reaction are affected by contextual variance [2]. While *B. recurrentis* infections were still common in European countries in the 19th century, including in the form of outbreaks in England, Scotland, and Ireland, Afro-Middle Eastern pandemics in the 20th century were followed by a residual area of endemicity in Ethiopia and its neighboring countries within the Horn of Africa [22,23,24,25,26].

*Bartonella quintana*, the causative agent of trench fever, has been associated with disease activity that largely depends on the immune status and the socio-economic status of the patients. Next to individuals infected with the human immunodeficiency virus (HIV), homeless poor people, alcohol, and drug addicts as well as warfighters dwelling in unfavorable front conditions (as witnessed in World War I) are particularly associated with clinical disease. *B. quintana* infections can comprise a wide spectrum of asymptomatic courses, but also relapsing fever (caused by a periodic bacteremia), endocarditis in HIV-seronegative individuals, and bacillary angiomatosis in immunosuppressed (e.g., AIDS) patients have been described [27,28,29,30,31,32]. Relapses are known to occur after short-course antibiotic therapy [28]. Bactericidal effects on *B. quintana* have been described for aminoglycosides [28]. In addition, tetracyclines, macrolides, and third-generation cephalosporins have been reported as therapeutically active [29]. An animal reservoir is unknown [33], but transmission of *B. quintana* via both head and body lice is considered a well-established route [34].

*Rickettsia prowazekii*, the causative agent of epidemic typhus, can also be spread if louse populations proliferate under poor sanitary conditions. The persistence of this agent is facilitated by delayed disease relapses, called Brill–Zinsser disease, and its natural reservoir host, the flying squirrel (*Glaucomys volans*) [35,36]. The failure of doxycycline prophylaxis for infection prevention has been reported, in spite of the fact that this antimicrobial serves as the therapy of choice in case of infections [37]. Lately, typhus outbreaks have predominantly been reported to occur in jails and refugee camps, particularly in resource-limited countries [4].

As reviewed in detail elsewhere [38], xenosurveillance in line with the “flying biological syringe” hypothesis constitutes a cost-efficient mode of surveillance for biological agents with relevance for human infections within their natural vectors. In the study presented here, we demonstrated this concept by screening *Pediculus humanus* lice collected from the clothes of Ethiopian homeless individuals for *Bartonella* species and *Borrelia* species.

## 2. Materials and Methods

### 2.1. Sample Collection

The sample collection was organized by the Hirsch Institute of Tropical Medicine, which is part of the University Hospital Düsseldorf and located in the Asella referral and teaching hospital compound in Ethiopia. We conducted a program in which new clothes were provided to 30 local homeless individuals under 20 years of age for humanitarian purposes on a voluntary basis in Asella, Ethiopia. Their original clothes were cleaned via autoclaving; prior to this procedure, body lice were collected from the clothes for subsequent molecular assessments. No association between collected lice and participating individuals was recorded to ensure absolute anonymity.

### 2.2. Molecular Diagnostics

The collected lice were stored and transported in 70% ethanol. Preparation was conducted with single-use consumables in order to avoid cross-contamination. Genomic nucleic acid was extracted via a Maxwell^®^ 16 device using a Maxwell LED DNA kit (Promega, Germany) as described in [39]. A 16S rRNA gene-specific pan-eubacterial PCR was initially performed [40] (Appendix Table A1 for details). Furthermore, *Bartonella* genus 16S-23S internal transcribed spacer (ITS) sequence-specific nested PCR [41] (Appendix Table A2 for details) and *Bartonella* genus *gltA* gene-specific nested PCR [42] (Appendix Table A3 for details) were conducted. Also, a 16S rRNA-gene PCR targeting relapsing-fever-associated *Borrelia* spp. was performed [43] (Appendix Table A4 for details). Amplicons with the expected sequence length were subjected to Sanger sequencing at Eurofins Scientific (Luxembourg). While results of the pan-eubacterial PCR were considered as preliminary only, any *Bartonella* spp. and *Borrelia* spp. sequences obtained with the *Bartonella*-genus- and *Borrelia*-genus-specific primers were deposited at NCBI GenBank. All laboratory assessments were conducted in line with standard requirements for molecular diagnostic assessments in laboratory infrastructure accredited according to strictly controlled DIN EN ISO 17025 standards (certificate number D–PL–19082-02-04) [44].

### 2.3. Ethical Clearance

Ethical clearance was not applicable because no human patients or human-patient-related data were included in the assessment.

## 3. Results

A total of 23 *P. humanus* lice were subjected to molecular assessment. A total of 16 positive PCR signals were obtained via pan-eubacterial 16S rRNA gene PCR screening. From the 16 amplicons, DNA was extracted for sequencing from 13 samples with expected amplicon lengths. From a total of eight interpretable sequences, the endosymbiont *Candidatus* Riesia pediculicola was identified in seven instances, and *Bartonella quintana* (99% query coverage, 99% sequence identity) was identified in one instance, albeit with relatively poor sequence quality as defined by the ambiguous signals in the trace data from Sanger DNA sequencing.

**Figure A1:**
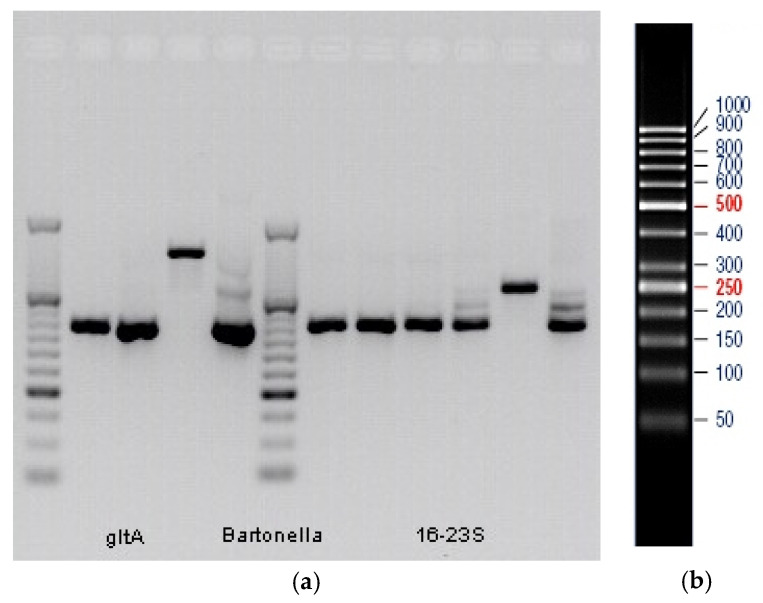
(**a**) Electrophoretic separation of positive amplification products of the *Bartonella* spp.-specific PCRs. Amplicons, from which *Bartonella* spp.-specific sequences could not be confirmed via Sanger sequencing, are shown as well. First and sixth columns: DNA mass standards. From second to fourth columns: first round of the Bartonella spp.-specific *gltA* gene PCR (356 bp amplicons in columns two and three, and 694 bp amplicon in column four). Fifth column: second round of the *Bartonella* spp.-specific *gltA* gene PCR (356 bp amplicon). Seventh to eleventh columns: first round of the *Bartonella* spp.-specific 16S-23S rRNA gene PCR (400 bp amplicons in columns seven to ten, and 539 bp amplicon in column eleven). Twelfth column: second round of the *Bartonella* spp.-specific 16S-23S rRNA gene PCR (400 bp amplicon). Sample HL10: second and ninth columns. Sample BL06: third and tenth columns. Sample BL12: fourth, fifth, eleventh, and twelfth columns. Sample L03: seventh column. Sample L05: eighth column. (**b**) Pattern of the applied DNA standard. Numbers indicate base pair (bp) counts.

In the same sample, the *Bartonella* genus 16S-23S internal transcribed spacer (ITS) sequence-specific PCR showed a positive result. The associated *B. quintana* sequence was deposited with the NCBI GenBank accession number OR504472. Once more from this sample and from two others as well, three *B. quintana gltA* sequences were obtained and deposited at NCBI GenBank. The associated accession numbers are OR499104, OR499105, and OR499106. A photograph of the electrophoretic separation of positive amplification products, also comprising amplicons from which *Bartonella* spp.-specific sequences could not be confirmed via Sanger sequencing, are provided in Appendix A (Figure A1).

Three positive signals were seen using the PCR targeting the 16S rRNA locus of relapsing-fever-associated *Borrelia* spp. Interpretable sequences were obtained from two out of the three amplicons corresponding to the endosymbiont *Candidatus* Riesia pediculicola. *Borrelia* spp.-specific sequences were not recorded.

## 4. Discussion

The limited xenosurveillance approach applied indicated the presence of *Bartonella quintana* in three out of twenty-three (13.0%) body lice collected from the clothes of homeless individuals in Ethiopia. The occurrence of *B. quintana* in Ethiopian lice and patients has been repeatedly described [45,46,47,48]. Insofar, the results observed in this assessment are not surprising. However, the fact that positive findings were achieved even with a very low number of assessed *P. humanus* lice indicates either random matching or, more likely, a substantial infection pressure for lice-infested homeless local individuals. Due to the design of the study reported here, it remains unknown whether any of the individuals whose clothes were assessed showed any clinical symptoms. However, due to the heterogenicity of symptoms in *B. quintana* infections [27,28,29,30,31,32], even a lack of symptoms would not have excluded an infection.

As the endemicity of *B. recurrentis* in Ethiopia is considered well established [22,23,24,25,26], a *Borrelia*-specific PCR was added to the pan-bacterial 16S rRNA gene PCR. However, both approaches failed to provide a *Borrelia*-specific sequence. Additionally, *Rickettsia prowazekii* DNA was not detected in any of the lice.

This study has a number of limitations. The low number of investigated lice provided only superficial insight into the local epidemiology of the lice-transmitted agents of infectious diseases. To provide more reliable data in a cross-sectional assessment, a larger sample size would be necessary. Collecting such a sample was, unfortunately, unfeasible due to financial restraints in this investigator-initiated assessment. Finally, the fact that the cultivation of *Bartonella quintana* was not attempted in this assessment must be considered a limitation.

## 5. Conclusions

In spite of the above-mentioned limitations, the described xenosurveillance approach once more confirmed that *B. quintana* is an infection risk for lice-infested individuals in Ethiopia. This finding stresses the need for further *Bartonella* spp. surveillance and for providing basic hygiene options for local homeless individuals for infection control and prevention purposes. Also, *Bartonalla* spp. infection needs to be considered in migrants from Ethiopia. Such infections can both endanger migrants’ health and contribute to the spread of bacterial pathogens in the host countries.

## Data Availability

All relevant data have been provided within the manuscript. Raw data can be made available at reasonable request.

## Appendix A

**Table A1:** Details of the 16S rRNA gene-specific eubacterial screening PCR.

	16S rRNA Gene-Specific Pan-Eubacterial PCR
Forward primer	27F (5′-AGAGTTTGATCMTGGCTCAG-3′)
Reverse primer	1392r (5′-ACGGGCGGTGTGTRC-3′)
Reaction mix	30 µL volumes containing 15 µL of Qiagen (Hilden, Germany) Hot Start Mix, 6 µL of PCR-grade H_2_O, 3 µL of each primer (final concentration 10 pmol), 5 µL of DNA eluate
Initial denaturation	15 min at 95 °C
Denaturation	30 s at 94 °C
Annealing	30 s at 54 °C
Amplification	1.5 min at 72 °C
Cycle numbers	30 cycles
Final extension	7 min at 72 °C
Gel electrophoresis	Application of 6µL PCR product + 2 µL loading dye to a 1.5% agarose gel with 0.065% Gel Red nucleic acid stain

**Table A2:** Details of the *Bartonella* genus 16S-23S internal transcribed spacer (ITS) sequence-specific, two-round PCR.

	*Bartonella* Genus 16S-23S-ITS-Specific PCR (First Round)	*Bartonella* Genus 16S-23S-ITS-Specific PCR (Second Round)
Forward primer	new-bigF (5′-GGAAGGTTTTCCGGTTTATC-3′)	new-bigF (5′-GGAAGGTTTTCCGGTTTATC-3′)
Reverse primer	new-bogR (5′-GTCTGAATATA(C/T)CTTCTCTTCAC-3′)	bigR (5′-TCCCAGCTGAGCTACG-3′)
Reaction mix	25 µL volumes containing 12.5 µL of Qiagen (Hilden, Germany) Hot Start Mix, 9.5 µL of PCR-grade H_2_O, 1 µL of each primer (final concentration 10 pmol), 1 µL of DNA eluate	
Initial denaturation	15 min at 95 °C	
Denaturation	30 s at 95 °C	
Annealing	30 s at 55 °C	
Amplification	1.0 min at 72 °C	
Cycle numbers	35 cycles	
Final extension	5 min at 72 °C	
Gel electrophoresis	Application of 6 µL PCR product + 2 µL loading dye to a 1.5% agarose gel with 0.065% Gel Red nucleic acid stain	

**Table A3:** Details of the *Bartonella* genus *gltA* gene-specific, two-round PCR.

	*Bartonella* Genus *gltA* Gene, First Round	*Bartonella* Genus *gltA* Gene, Second Round
Forward primer	443f (5′-GCTATGTCTGCATTCTATCA-3′)	781f (5′-GGGGACCAGCTCATGGTGG-3′)
Reverse primer	1137r (5′-AATGCAAAAAGAACAGTAAACA-3′)	1137r (5′-AATGCAAAAAGAACAGTAAACA-3′)
Reaction mix	50 µL volumes containing 25 µL of Qiagen (Hilden, Germany) Hot Start Mix, 20 µL of PCR-grade H_2_O, 2 µL of each primer (final concentration 10 pmol), 1 µL of DNA eluate	
Initial denaturation	15 min at 95 °C	
Denaturation	30 s at 95 °C	
Annealing	30 s at 55 °C	
Amplification	1.0 min at 72 °C	
Cycle numbers	35 cycles	
Final extension	5 min at 72 °C	
Gel electrophoresis	Application of 6µL PCR product + 2 µL loading dye to a 1.5% agarose gel with 0.065% Gel Red nucleic acid stain	

**Table A4:** Details of PCR targeting relapsing-fever-associated *Borrelia* spp.

	16S rRNA Gene PCR Targeting Relapsing-Fever-Associated *Borrelia* spp.
Forward primer	5′-GGCTTAGAACTAACGCTGGCAGTGC-3′
Reverse primer	5′-CCCTTTACGCCCAATAATCCCGA-3′
Reaction mix	30 µL volumes containing 15 µL of Qiagen (Hilden, Germany) Hot Start Mix, 6 µL of PCR-grade H_2_O, 3 µL of each primer (final concentration 10 pmol), 5 µL of DNA eluate
Initial denaturation	15 min at 95 °C
Denaturation	15 s at 94 °C
Annealing	30 s at 54 °C
Amplification	1 min at 72 °C
Cycle numbers	40 cycles
Final extension	7 min at 72 °C
Gel electrophoresis	Application of 6µL PCR product + 2 µL loading dye to a 1.5% agarose gel with 0.065% Gel Red nucleic acid stain
